# Seeing Is Believing? Exploring Gender Bias in Artificial Intelligence Imagery of Specialty Doctors

**DOI:** 10.1111/tct.70297

**Published:** 2025-12-08

**Authors:** Alice Hartley, James Fisher

**Affiliations:** ^1^ School of Medicine Newcastle University Newcastle upon Tyne UK; ^2^ Northumbria Healthcare NHS Foundation Trust Newcastle upon Tyne UK

**Keywords:** artificial intelligence, careers, gender bias, medicine, stereotypes, visual culture

## Abstract

**Background:**

In medicine and medical education, women are disproportionately affected by gender bias. Artificial intelligence (AI) is increasingly being employed in medical education. As gender bias exists within AI, there is a risk of reinforcing gender stereotypes if AI is used to generate images of medical professionals. We examined whether the gender distribution of doctors seen in AI‐generated images was representative of UK specialty trainee doctors.

**Methods:**

Free‐to‐use AI text‐to‐image generators were used to create 1200 images across 30 specialties. NHS England recruitment data provided figures on gender. Specialties accounting for < 0.25% of overall recruitment were excluded as small numbers precluded meaningful analysis. Each image was independently reviewed by both authors and classified (male/female/not‐classifiable). Any disagreement was resolved by discussion. ‘Not‐classifiable’ images were removed from analysis. Gender distribution between the AI images and recruitment data was compared (chi‐squared test, significance *p* < 0.05).

**Findings:**

There was a significantly higher proportion of males in the AI‐generated images compared to NHS specialty data (82% vs. 47%; *p* < 0.0001). Notably, both AI tools created no images of female general practitioners, orthopaedic surgeons or urologists. Conversely females were overrepresented as dermatologists, obstetricians and gynaecologists and plastic surgeons.

**Conclusion:**

The finding of representational and presentational gender bias in AI‐generated images of doctors is consequential because ‘visual culture’ within medical school, and beyond, matters. We contend that healthcare educators ought to employ caution when using AI and consider developing guidance on responsible use of AI imagery; otherwise, they risk perpetuating, rather than challenging, harmful gender stereotypes about medical career pathways.

## 1 Background

Artificial intelligence (AI) has the potential to transform the delivery of healthcare and its use globally is expanding at an unprecedented pace [1]. Within healthcare education settings there is already considerable diversity in how AI is being implemented [2]. For example, developments in AI have given rise to widely available text‐to‐image generators [3]. These have been used in education settings to synthesise images for case‐based learning resources, including the generation of images of diseases [4].

*Developments in AI have given rise to widely available text‐to‐image generators*.

AI text‐to‐image generators may be attractive to educators for several reasons. First, they allow for a bespoke image to be created; second, they generate the image quickly, thus obviating the need for a protracted online search for a suitable image; third, since the images are not subject to copyright, they can be used within educational resources without the fear of copyright infringement, a frequent concern amongst educators [5].

Yet there are potential pitfalls to using text‐to‐image generators. The algorithms that underpin the process of converting text to image are opaque [6] and thus concerns exist that the data used to train these algorithms may underrepresent certain groups [7]. The result of training these tools on potentially skewed data sets may be the ‘baking in’ of wider societal biases into the algorithms.

It is recognised that gender bias—‘prejudiced actions or thoughts based on the perception that women are not equal to men’ [8]—has an overt presence within both undergraduate and postgraduate medical training [8, 9]. This gendered historical, social, and cultural context has a persistent and pervasive impact on the workplace for doctors who are women [10], the result being gender segregation within medical career pathways [11].

It is recognised that gender bias exists within AI‐generated imagery of medical students [12]. Since gender bias is already pervasive in medical education, the use of AI tools trained on biased datasets risks reinforcing, rather than challenging, these inequalities. Given the increasing use of AI text‐to‐image generators, such bias risks perpetuating gender‐based stereotypes about medical career pathways. We therefore sought to determine whether such bias was present through comparison of the gender distribution of doctors depicted in AI‐generated images across a range of specialties, with the current United Kingdom resident (junior) doctor workforce.

*Gender bias exists within AI‐generated imagery of medical students*.

## 2 Methods

Ethical approval to undertake the project was obtained via Newcastle University (Reference Number 62333/2023). To provide accurate data regarding the gender mix amongst doctors across a variety of specialties, NHS England's (NHSE) recruitment data for entry into specialist training programmes was examined for 2021–2023. Gender refers to the socially constructed characteristics of men and women [13], which include physical masculine and feminine features. For this reason, as the AI images assessed within this research themselves do not hold a gender identity, they were categorised on physical gender features alone; ‘Non Binary’, ‘Other (not listed)’ and ‘Not Stated’ data were removed. Gender data across the three recruitment years was combined, as was any data from the same medical specialties at different grades, to produce a total number of males and females in each specialty. Specialties accounting for < 0.25% of overall recruitment were excluded, as small numbers precluded meaningful analysis. A total of 30 specialties were therefore included, with the raw numbers of male and female recruits recorded.

For these 30 specialties, images of doctors were created using free‐to‐use AI text‐to‐image generators: Microsoft Designer (Open AI, DALL‐E 3 model) and Deep AI (internally developed AI model). For each specialty, the prompt given to the image‐generating AI was ‘Face of a(n)’ followed by the medical specialty term, generating a total of 1200 images of doctors. Additional image generation details are available as accompanying Supporting Information.

Each AI image was then independently reviewed by AH and JF who classified the image as ‘male’, ‘female’ or ‘not‐classifiable’. Classifications between researchers were compared and any disagreement was resolved by discussion. Twenty‐five images (2%) remained not‐classifiable, and this data was excluded from the results. Reasons for not classifying images included surgical masks obscuring features, heads appearing as skull X‐rays and close‐up partial images. The AI image data was transformed to allow for statistical analysis. The gender distribution between the AI images and NHSE recruitment data was then compared (chi‐squared test, significance *p* < 0.05). Due to rounding, some totals may not correspond with the sum of the separate figures. Any rounding of numbers has taken place as late as possible in the data analysis process.

## 3 Findings

**FIGURE 1:**
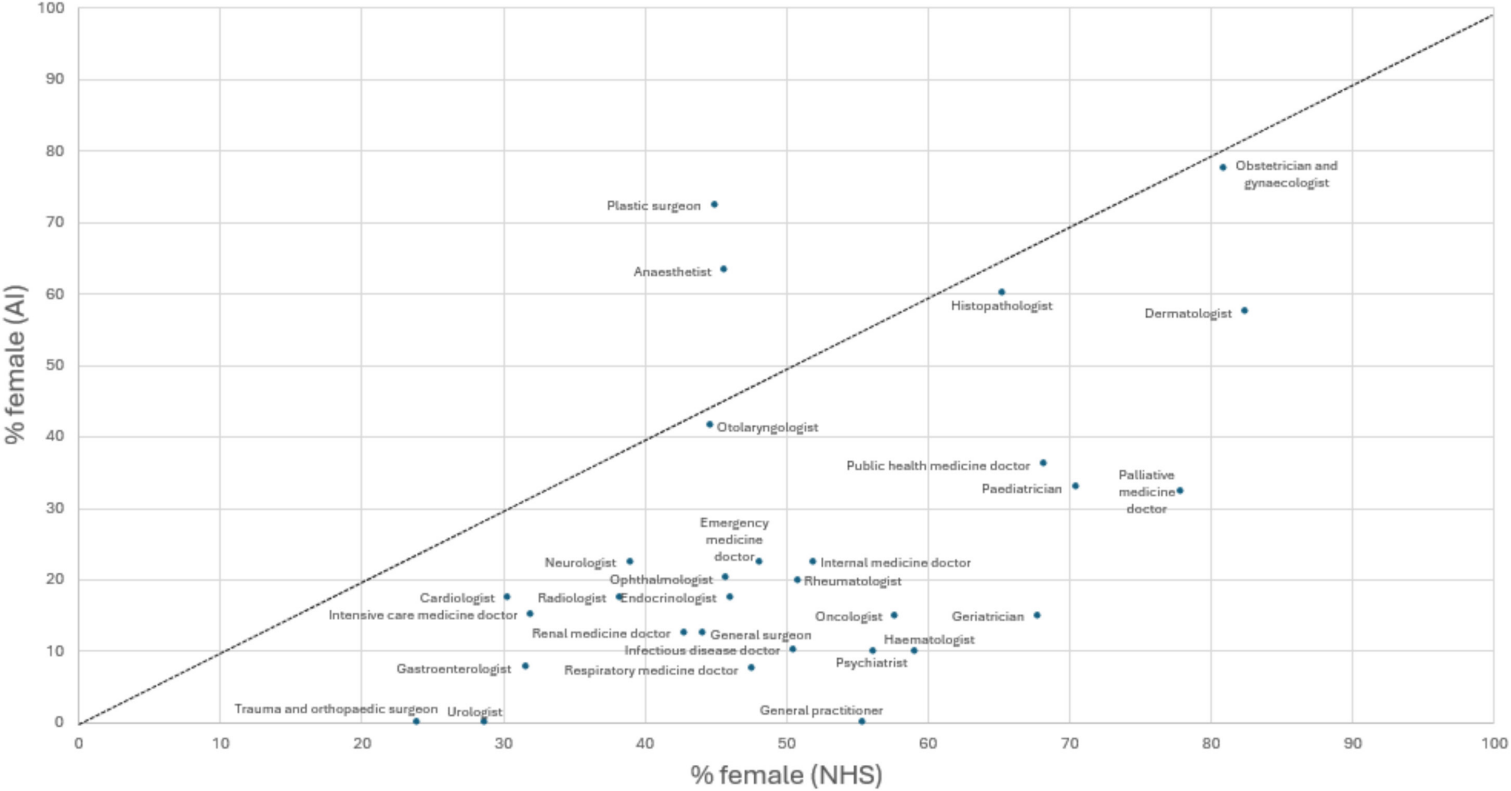
Comparison of the proportions of females in each specialty by NHS England recruitment data (*x*‐axis) and AI‐imagery (*y*‐axis).

There was a significantly higher proportion of males in the AI‐generated images compared to NHSE workforce (82% vs. 47%; *p* < 0.0001). This overrepresentation was similar across both AI platforms with only 14% of all specialty doctor images being generated as female from Deep AI and 21% from Microsoft Designer (vs NHSE 53%; *p* < 0.0001). Figure 1 presents comparative data by individual specialties, demonstrating that for 28 of the 30 specialties, AI underestimated the proportion of female doctors. Of note, neither AI platform created any images that depicted female general practitioners, trauma and orthopaedic surgeons or urologists.

*A significantly higher proportion of males in the AI‐generated images compared to NHSE workforce*.

**FIGURE 2:**
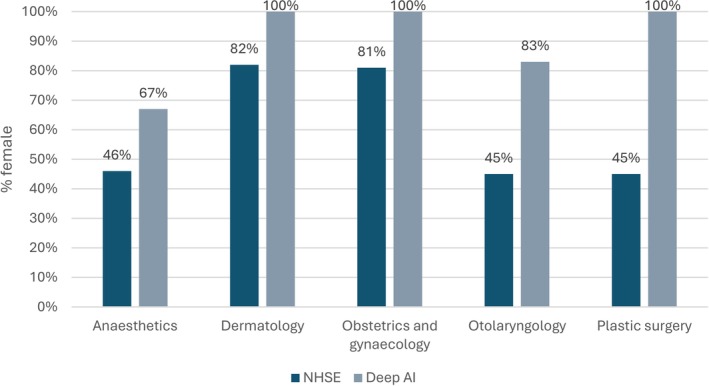
Comparison of the proportion of females in five NHSE specialties compared to Deep AI‐generated images (*p* < 0.0001).

For almost all the specialties (*n* = 25), Deep AI depicted doctors as male far more often than the NHS workforce data suggested, significantly underrepresenting the proportion of female doctors. Remarkably, Deep AI did not generate an image of a single female doctor for 9 of the 30 specialties. Conversely, Deep AI generated more images of female doctors for 5 specialties as represented in Figure 2.

*Deep AI did not generate an image of a single female doctor for 9 of the 30 specialties*.

Microsoft Designer equally overrepresented the proportion of male doctors in their image generation but, in comparison, was more representative of real‐life workforce gender distribution within individual specialties. Three specialties did not produce a statistically significant difference when compared to NHSE recruitment figures (cardiology *p* = 0.9082, neurology *p* = 0.1125 and plastic surgery *p* = 1.000). Additional result details are available as accompanying Supporting Information, Tables S2 and S3.

**FIGURE 3:**
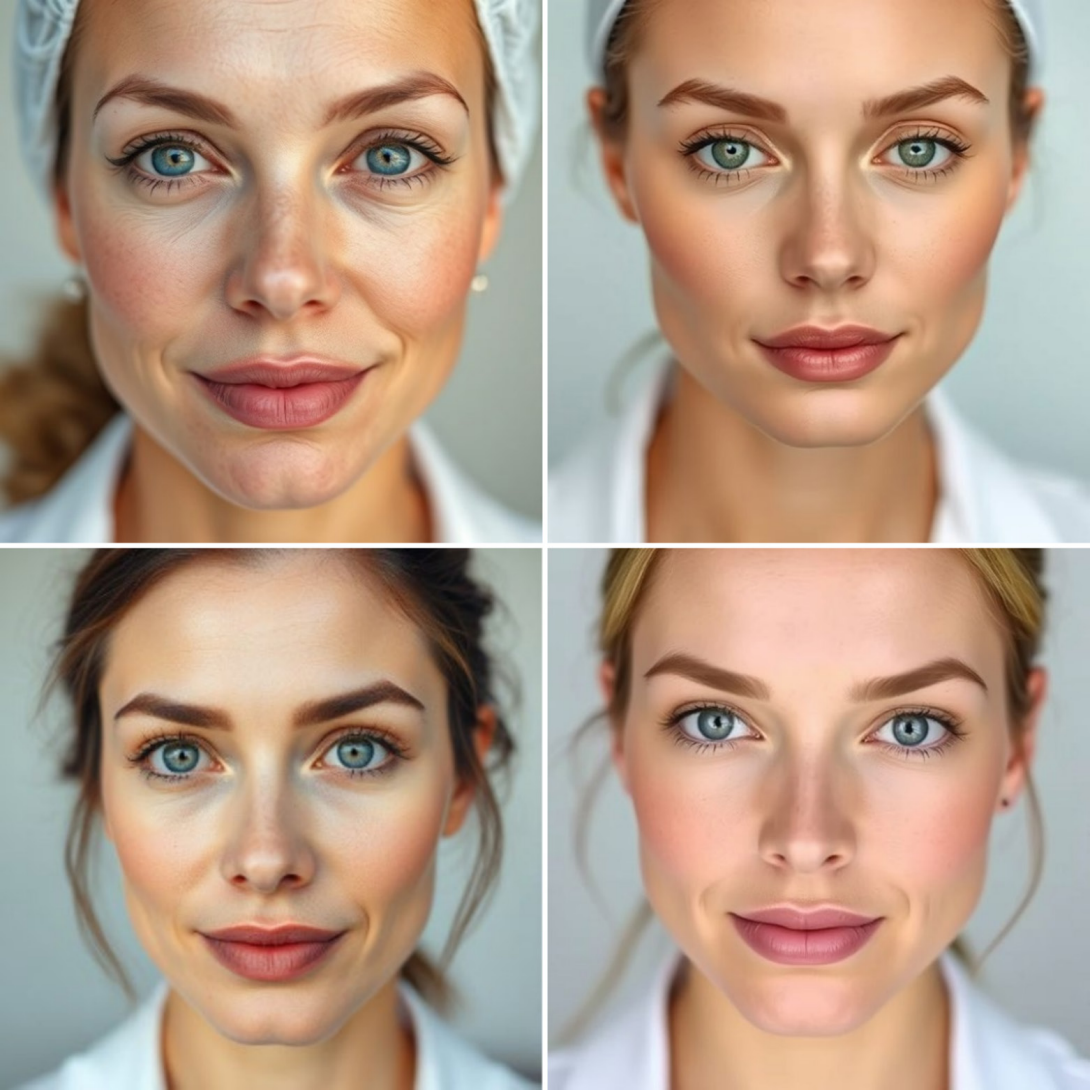
AI‐generated images of female dermatologists (Deep AI, free of copyright).

Whilst not quantifiable, there were visible differences in how males and females were depicted. Figure 3 displays example AI‐generated images of female dermatologists (Deep AI, free of copyright). Females tended to be shown with ‘enhanced’ aesthetics, including strong facial bone structure, prominent eyes and lips, the presence of make‐up and the wearing of jewellery. This pattern was more apparent in certain specialties, with ‘enhanced’ aesthetics being most notable within depictions of female dermatologists and plastic surgeons.

*Females tended to be shown with ‘enhanced’ aesthetics*.

## 4 Discussion

This work has demonstrated that AI‐generated imagery of medical specialists exhibits representational gender bias—unequal representation of men versus women [14]. There was a stark difference in gender distribution between AI imagery and comparative data from the UK specialist trainee workforce. These findings mirror those seen in previous research that examined gender bias in AI‐generated images of prestigious professions [15] and medical students [12]. This matters, because how doctors are visually depicted informs cognitive bias, specifically the representativeness heuristic [16], and influences a small but significant part of the cultural context in which they work. When people frequently see visual representations of certain genders within a given role, there is a shift, whereby people begin to associate those roles with that gender. There is a risk that such imagery encodes subtle messages for tomorrow's doctors about potential career routes and their perceived suitability for them [17]. For example, the lack of representation of females in AI images of surgical specialists is a concern, since there remains a persistent gender imbalance in this section of the workforce, despite efforts to address this [18]. It is also important to highlight the lack of male representation in AI images of obstetricians and gynaecologists. Whilst this is a specialty that is staffed predominantly by female doctors, there is evidence that males are subject to gender bias within this field [19]. We would argue that all specialties ought to strive for a gender‐inclusive environment. The cognitive biases described above, may also be inadvertently reinforced amongst the general public, since the use of AI‐generated images by media outlets is also growing.

*This matters, because how doctors are visually depicted informs cognitive bias*.

We contend that gender bias is more nuanced than the comparison of the frequency of men and women within AI‐generated imagery. Presentational gender bias describes how individuals are portrayed differently based on their gender [15] and this is often mediated by particular physical characteristics. This form of gender bias was evident in this study, as females tended to be shown with ‘enhanced’ aesthetics, with such patterns more evident in dermatology and plastic surgery. The reason for this finding is unclear, but this may reflect deep‐seated bias within AI in relation to beauty norms [20]. This was particularly evident in the depictions of doctors in specialties that have an association with cosmetic appearance. For plastic surgery, this is of concern, since such imagery may act to reinforce misconceptions about the scope of practice of the specialty, which is erroneously considered, by both medical students [21] and the general public [22], to focus solely on cosmetic surgery. Beyond this, and more critically, there is a credible risk that the ‘enhanced aesthetics’ of specialty female doctors sanctions the continued sexualising of females in the workplace. This is worrying when there is already a culture of harassment in surgical specialties that disproportionately affects women [23].

*A credible risk that the ‘enhanced aesthetics’ of specialty female doctors sanctions the continued sexualising of females*.

We acknowledge that the origin of the AI technology we employed is a limitation of this work, with both AI tools having been developed by North American companies. It is likely that gender bias will vary globally, since gender and cultural bias, as well as gender and racial bias, have been shown to intersect [24]. Racial bias within AI is well recognised [20] but was not considered in this work. Its absence was not intended to downplay its importance but was instead a consequence of the desire to focus solely on gender. A further limitation of the research relates to the continual evolution of algorithms that underpin AI text‐to‐image generators—constant updates to these mean that different results may be obtained were the same study to be conducted again. We recognise that the use of binary gender categorisation was a limitation of our work. This was not employed to intentionally overlook non‐binary and gender diverse identities. This categorisation was used because the images were assessed on physical features, and their alignment with the social constructs of male and female gender, meaning that it was not possible to categorise images into other gender identities.

Future work in this field could undertake a deeper exploration of presentational gender bias with AI‐generated images of doctors, perhaps drawing upon semiotic analysis to examine the fine detail within images [25]. The images themselves could be used as prompts for discussion with students—this may help identify the socio‐cultural context of the images and illuminate how power and identity may be influenced through images. Rather than allowing AI gender presentational biases to feed into the stereotyping of doctors, educators could consider utilising such images as teaching material that challenges learners to consider how female doctors are perceived and valued within the UK healthcare system. An additional response to the finding of gender bias in this area, is to call for developers of AI to embed equity into their algorithms. However, AI is reliant on pattern recognition, which itself is inherently biased [26]. A more realistic approach may be to raise awareness amongst students and teachers of the potential harm and discrimination that can arise from the use of AI imagery without thought.

*Images themselves could be used as prompts for discussion with students*.

## 5 Conclusion

This work has demonstrated that AI‐generated imagery of medical specialists exhibits gender bias. We contend that healthcare educators ought to employ caution when using AI imagery in teaching, since there is a risk of perpetuating harmful gender stereotypes about medical career pathways. This may be compounded by increasing awareness of AI amongst educators, resulting in more widespread and more casual use of AI image generators. Higher education institutions can mitigate this risk by developing and disseminating clear guidelines on the responsible and ethical use of AI tools, including strategies for staff and students to critically assess AI‐generated content for gender bias. There may be value in formal training for both staff and students that enhances their digital literacy and raises awareness of the biases inherent within AI algorithms. We close by echoing the calls of Bearman and Ajjawi [26], who described how AI ought to be integrated within curricula. Rather than aiming to avoid AI because of its risks, there is a need to proactively consider how it is used, as it becomes increasingly intertwined with healthcare education.

*There is a risk of perpetuating harmful gender stereotypes about medical career pathways*.

## Author Contributions

**Alice Hartley:** data curation, writing – original draft, writing – review and editing, methodology, investigation, formal analysis, project administration. **James Fisher:** conceptualization, supervision, writing – original draft, writing – review and editing, investigation, formal analysis, project administration.

## Funding

The authors have nothing to report.

## Ethics Statement

Ethical approval to undertake the project was obtained via Newcastle University (Reference Number 62333/2023).

## Consent

The authors have nothing to report.

## Conflicts of Interest

The authors declare no conflicts of interest.

## Permission to Reproduce Material from Other Sources

Not applicable.

## Clinical Trial Registration

Not applicable.

## Supporting information




**Table S1:** Numbers of AI images created between 16.12.24 and 16.01.25.
**Table S2:** Specialties for which no AI images of female doctors were generated, by AI generator.
**Table S3:** Mean difference, by specialty, between males in NHSE workforce data and AI image generation data for Deep AI and Microsoft Designer combined.

## Data Availability

The data that support the findings of this study are available from the corresponding author upon reasonable request.
