# Supplementary material for: Seeing Is Believing? Exploring Gender Bias in Artificial Intelligence Imagery of Specialty Doctors
Source: Clin Teach. 2025 Dec 8;23(1):e70297. doi: 10.1111/tct.70297 (PMC12685619; doi:10.1111/tct.70297)
Supplement: Supplementary file 1 — Table S1: Numbers of AI images created between 16.12.24 and 16.01.25. Table S2: Specialties for which no AI images of female doctors were generated, by AI generator. Table S3: Mean difference, by specialty, between males in NHSE workforce data and AI image generation data for Deep AI and Microsoft Designer combined. [file TCT-23-e70297-s001.docx]

**Supplementary material**

*Table S1: Numbers of AI images created between 16.12.24 and 16.01.25*

| **Healthcare specialty** | **AI generator search term**  **“Face of a(n)..”** | **Number of images generated** | |
| --- | --- | --- | --- |
|  |  | **Deep AI** | **Microsoft Designer** |
| Anaesthetics | Anaesthetist | 20 | 20 |
| Emergency medicine | Emergency medicine doctor | 20 | 20 |
| Internal medicine | Internal medicine doctor | 20 | 20 |
| Cardiology | Cardiologist | 20 | 20 |
| Clinical radiology | Radiologist | 20 | 20 |
| Combined infection training | Infectious disease doctor | 20 | 20 |
| Core psychiatry | Psychiatrist | 20 | 20 |
| Dermatology | Dermatologist | 20 | 20 |
| Endocrinology and diabetes mellitus | Endocrinologist | 20 | 20 |
| Gastroenterology | Gastroenterologist | 20 | 20 |
| General practice | General practitioner | 20 | 20 |
| General surgery | General surgeon | 20 | 20 |
| Geriatric medicine | Geriatrician | 20 | 20 |
| Haematology | Haematologist | 20 | 20 |
| Histopathology | Histopathologist | 20 | 20 |
| Intensive care medicine | Intensive care medicine doctor | 20 | 20 |
| Neurology | Neurologist | 20 | 20 |
| Obstetrics and gynaecology | Obstetrician and gynaecologist | 20 | 20 |
| Oncology (clinical and medical) | Oncologist | 20 | 20 |
| Ophthalmology | Ophthalmologist | 20 | 20 |
| Otolaryngology | Otolaryngologist | 20 | 20 |
| Paediatrics | Paediatrician | 20 | 20 |
| Palliative medicine | Palliative medicine doctor | 20 | 20 |
| Plastic surgery | Plastic surgeon | 20 | 20 |
| Public health medicine | Public health medicine doctor | 20 | 20 |
| Renal medicine | Renal medicine doctor | 20 | 20 |
| Respiratory medicine | Respiratory medicine doctor | 20 | 20 |
| Rheumatology | Rheumatologist | 20 | 20 |
| Trauma and orthopaedic surgery | Trauma and orthopaedic surgeon | 20 | 20 |
| Urology | Urologist | 20 | 20 |

*Table S2: Specialties for which no AI images of female doctors were generated, by AI generator.*

| **Deep AI** | **Microsoft Designer** |
| --- | --- |
| Emergency medicine | Anaesthetics |
| Haematology | General practice |
| General practice | Otolaryngology |
| Neurology | Trauma and orthopaedics |
| Psychiatry | Urology |
| Radiology |  |
| Renal medicine |  |
| Trauma and orthopaedics |  |
| Urology |  |

*Table S3: Mean difference, by specialty, between males in NHSE workforce data and AI image generation data for Deep AI and Microsoft Designer combined.*

| **Specialty** | **% difference in males** |
| --- | --- |
| Plastic surgeon | -28 |
| Anaesthetist | -18 |
| Otolaryngologist | 3 |
| Obstetrician and gynaecologist | 4 |
| Histopathologist | 5 |
| Cardiologist | 13 |
| Intensive care medicine doctor | 17 |
| Neurologist | 17 |
| Radiologist | 21 |
| Gastroenterologist | 24 |
| Trauma and orthopaedic surgeon | 24 |
| Dermatologist | 25 |
| Emergency medicine doctor | 26 |
| Ophthalmologist | 26 |
| Endocrinologist | 29 |
| Urologist | 29 |
| Internal medicine doctor | 30 |
| Renal medicine doctor | 31 |
| Rheumatologist | 31 |
| General surgeon | 32 |
| Public health medicine doctor | 32 |
| Paediatrician | 37 |
| Respiratory medicine doctor | 41 |
| Infectious disease doctor | 41 |
| Oncologist | 43 |
| Palliative medicine doctor | 46 |
| Psychiatrist | 47 |
| Haematologist | 49 |
| Geriatrician | 53 |
| General practitioner | 55 |
| Overall | 36 |
